# Let Us Not Forget the Victims of COVID-19 Pandemics Who Did Not Die With the Coronavirus

**DOI:** 10.3389/fpubh.2022.900100

**Published:** 2022-05-09

**Authors:** Anton Pashkevich, Tomasz E. Burghardt

**Affiliations:** ^1^Faculty of Civil Engineering, Politechnika Krakowska, Kraków, Poland; ^2^M. Swarovski Gesellschaft m.b.H., Neufurth, Austria

**Keywords:** excess mortality, pandemic impact, non-COVID mortality, health care denial, underreporting

On 11 March 2020 the World Health Organisation (WHO) declared COVID-19 a pandemic. As of 31 December 2021, worldwide there were reported 288,702,042 cases and 5,436,385 fatalities amongst those who were diagnosed with the disease (1). A search in SCOPUS database for “COVID-19” revealed 249,437 scholarly articles published until end of 2021; despite such amassed knowledge, the topic of mortality not associated with this disease remains very weakly represented, with very few published studies (2–4).

While searching for information related to road transportation safety in Poland, we have stumbled upon statistical data that left us flabbergasted: excess non-COVID mortality during the pandemic. According to weekly reports published by Statistics Poland (Główny Urzad Statystyczny, GUS), the total excess mortality in 2020 and 2021 was 228,308 over the average from 2010 to 2019 period (5). However, only 97,592 (42.7%) of those excess deaths occurred amongst people who were diagnosed with COVID-19 (1). This is better illustrated in Figure 1, where the weekly excess mortality is charted separately for the COVID-19 and non-COVID cases. A baffling trend could be observed: the peaks in non-COVID excess mortality occurred about 1 month earlier than surges in COVID-19 fatalities. The drops in non-COVID mortality to below the 10-year average can serve as a confirmation that the excessive deaths during the time of pandemics cannot be treated as a natural unrelated trend.

**Figure 1:**
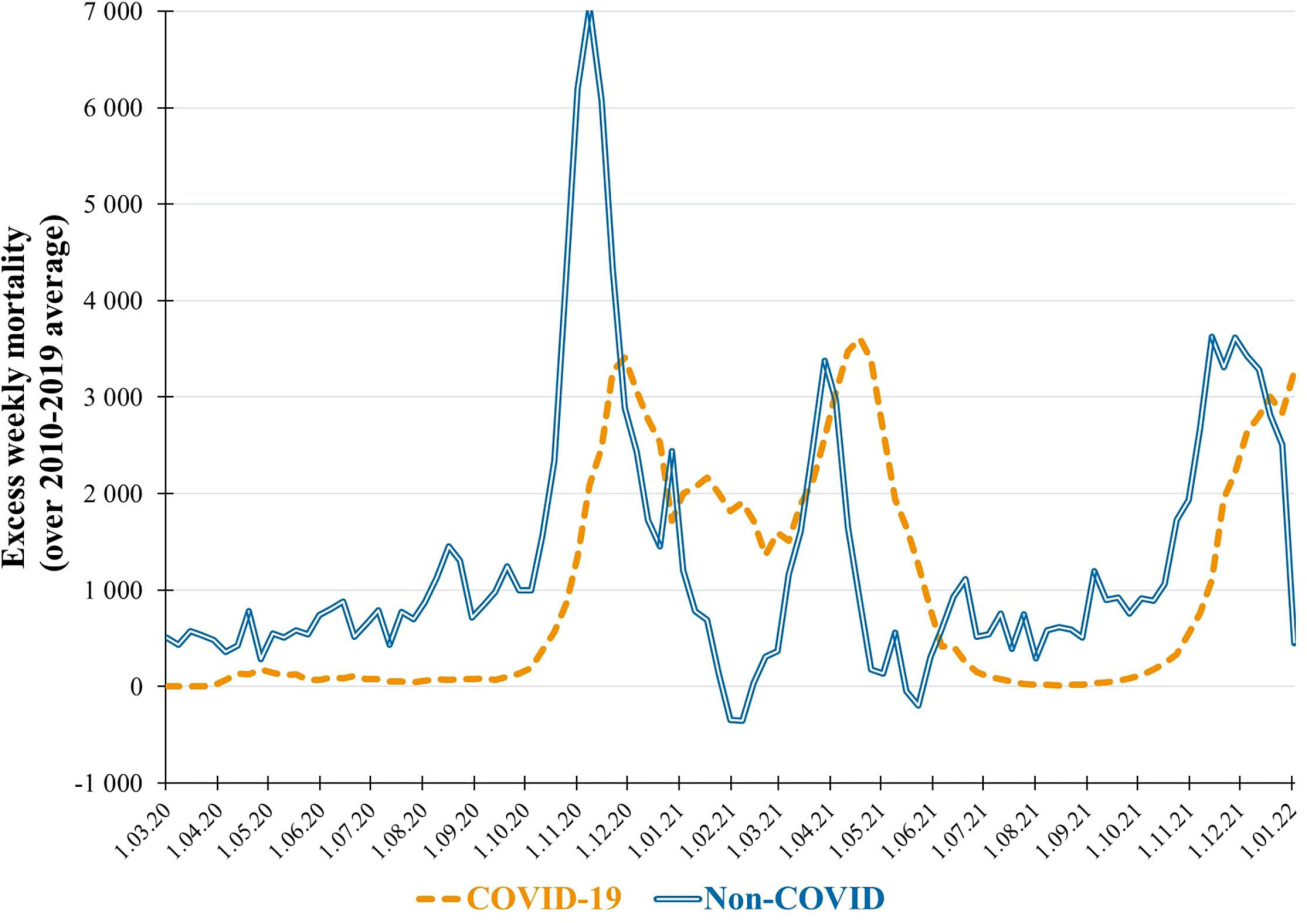
Weekly excess mortality over 2010−2019 average.

The purpose of this opinion letter is to alert the World's scientific community about the thousands of silent deaths of those who were very likely the victims of the disease despite not being diagnosed with it. There could be many contributing factors and their combination that caused such situation. It is quite unlikely that such enormous additional mortality could be due to underreporting of COVID-19, given that all people seeking help from medical professionals were tested; nonetheless, the mortality curves appear to be correlated and underestimation would not be unrealistic. Quite possibly, this effect was caused by excessive focus of public health on one ailment with neglect for other diseases that caused effective denial of service; this would mean that most of those people could be considered as “collateral damage.” Nonetheless, without extensive research, combining various perspectives, it is not possible to pinpoint the real causes, so feasible solutions and necessary structural changes of health care systems and the response to crises could be proposed. Because recognition of a problem is always the first step to solve it, we believe that this short note would suffice to direct some of the attention to this mostly ignored perspective.

## Author Contributions

AP and TB wrote the main text. TB prepared the Figure 1. All authors reviewed and accepted the manuscript.

## Conflict of Interest

TB was employed by M. Swarovski Gesellschaft m.b.H. The remaining author declares that the research was conducted in the absence of any commercial or financial relationships that could be construed as a potential conflict of interest.

## Publisher's Note

All claims expressed in this article are solely those of the authors and do not necessarily represent those of their affiliated organizations, or those of the publisher, the editors and the reviewers. Any product that may be evaluated in this article, or claim that may be made by its manufacturer, is not guaranteed or endorsed by the publisher.
